# Texas Fever (Southern Cattle Fever, Etc.)

**Published:** 1890-07

**Authors:** Paul Paquin

**Affiliations:** State Veterinarian, Missouri


### THE JOURNAL
OF
### COMPARATIVE MEDICINE AND VETERINARY ARCHIVES.

VOL. XI. JULY, 1890. NO. 7.

### TEXAS FEVER.
### (SOUTHERN CATTLE FEVER, ETC.)

BY PAUL PAQUIN,
*State Veterinarian, Missouri.*

No disease of cattle causes anything like as great a loss yearly to the people of the West and South as does this misnamed and fatal one, and not unfrequently it is carried even to the East. The Northern farmer and stockman is almost absolutely deprived of the grand market that the infectious Southern States offer to them. Fifty to seventy per cent. of the Northern cattle shipped South of the fever line including, of course, the importations of improved stock to raise the quality of the Southerner's herd, die from Texas fever. On the other hand the Northern pastures, that offer such rich source of profit to cattle feeders, are forbidden to the Southern cattle, both by the contagion which they carry and by laws passed in consequence. If there be transgression, several usually pay dearly for the sin by the death of cattle and lawsuits. Thus, the damages caused to all these States that must profit enormously by interstate cattle commerce, if it were not for that uncontrollable malady, are incalculable. Counting with those difficulties the legal and moral restrictions on commerce by quarantine regulations, the situation is indeed gloomy.

Arrangements were made with the Agricultural College Experiment Station for intershipments of experiment stock and cooperation in investigation—the direction of the studies to be with me. Later, like arrangements were made with a similar institution at Fayetteville, Arkansas. At about the same time I also arranged for researches in the Indian Territory, and then to experiment at some public stock yards in Missouri. Stock pens were also constructed for Southern cattle and others a few miles from my home in Columbia.

Before setting to work we formulated the following propositions:

1. Determine the places in the soils, waters, grasses, etc., of Texas and other places in which Texas fever virus exists.

2. Determine the part or parts in the body of Southern cattle, or their products, where Texas fever virus may be found (and from whence it may be transferred).

3. Determine the accessory sources (of transmission of virus) such as hay, ticks, hoofs, foods, etc.

4. Determine the period of incubation of the disease in Northern cattle.

5. Determine the duration of infection in Southern stock (*i. e.*, how long they are capable of conveying Texas fever).

6. Determine the best and cheapest modes of disinfection of cars, yards, etc.

7. Test means to disinfect Southern cattle alive and render them harmless before shipping North.

8. Experiment with a view to render Northern cattle safe against Texas fever before shipping them South—that is, to make them proof against Texas fever by vaccination or otherwise.

9. Determine whether horses or other animals ever take truly, or bring us Texas fever as seems to have occurred.

10. Determine how long on pastures or other places in the North, the Texas fever germs exist in dangerous nature.

11. Determine if under any conditions Northern cattle can cause infection of other Northern stock.

12. Determine whether calves born South become proof against Texas fever by natural inoculation from the mother before birth, or afterward on the soils, or if by both processes.

During my trip in Texas and the Indian Territory, September, 1888, I collected soils, manures, urines, ticks, livers, spleens, kidneys, blood, bile, specimens from unborn calves, fodders, and waters from various sources on infectious grounds. Later Dr. M. Francis, of the Agricultural College and Experiment

Station of Texas, furnished me with a great number of articles of the same order, and later still Dr. Dinwiddie, of Arkansas, gathered several. As a result, between September, 1888—*i. e.*, during the cool and cold seasons, we investigated at the laboratory 148 different specimens from the South—over 100 of which had been gathered under written instructions by Dr. Francis in counties of Texas, North, South, East and West.[1] Such specimens as liver, kidney, spleen, etc., were taken fresh immediately after death of the animals at slaughter houses, then wrapped in cloths previously soaked in either a strong solution of bichloride of mercury (corrosive sublimate), or carbolic acid; then they were immersed in pure glycerine in fruit jars and sealed. They all were preserved absolutely free from the usual results of contamination by air germs. The specimens of blood, bile and urine were nearly all sealed in sterilized glass tubes (pipettes) without being exposed to the air. Really all the foregoing substances, therefore, were preserved and studied containing the same things as before the death of the animals that furnished them.

Our endeavors were first directed toward the discovery in the foregoing specimens of a virus capable of producing Texas fever. With that end in view many difficult problems presented themselves. It was during the cold season when the disease is induced with difficulty, and at a period when we knew of no other animal susceptible to the fever in question than cattle, which could scarcely be bought for less than $20 per head in average. Our limited means precluded the use of many such comparatively high priced experiment subjects. Consequently we had to go very slowly.

*First Investigations:—Blood, Urine, Bile, Liver, Spleen and Kidney from Healthy Southern Cattle and from Unborn Calves,* (*fœtus*)—Thirty-three different specimens from twenty different Southern cattle of several counties of Texas and the Indian Territory were analyzed. In all of them without exception we found after a long and tedious search germs that could be seen plainly and stained fairly well. Unstained in the tissues of the liver, spleen, kidneys, glands, the germs appeared as bright or bronze-colored corpuscles, of irregular shapes, apparently grouped in and out of the cells of the organs, and disseminated in the spaces and tissues. Some of these bodies appeared almost spherical, others

[1] Dr. Dinwiddie's specimens were not sent that season.

like bright specks, and still others as ovoid bodies or short oval rods of various dimensions. In the blood and bile the germs were found frequently in a motile condition. They would roll on themselves, bend two and fro, move from place to place, taking a variety of shapes which gave them a resemblance with some of the corpuscles found in the liver, etc. The germs could be stained in the blood and tissues, but only with difficulty in the bile. The forms in the blood were small and like the finer organisms in the tissues. The blood elements were impaired. In the bile, the germs were often in the shape of rods of considerable length. It contained, also, as the kidneys and urine, fine, bright bodies, occasionally double as diplococci.

Our first attempts to cultivate these germs artificially were not satisfactory, but still we did reproduce some forms. Our next step was to investigate the soils, manures, waters, fodders, etc., to see if the same species of germs could be found. We discovered similar forms mingled with numerous others in every specimen with the exception of dry hay, subsoil, spring water, well water, and one specimen of surface soil. The manures were particularly prolific. We made cultures and succeeded with difficulty and not very satisfactorily to produce germs similar in appearance and in mode of growth to those grown from liver, spleen, kidney and bile. Inoculation of white mice with these cultures in state of purity caused death occasionally, but only when a large quantity was used. The same germs were found in the liver, spleen, etc., of the little animals that had thus perished. With these cultures, however, we could not kill cattle with typical symptoms of Texas fever, although we did produce fever as high as 104° and 105° F. in some of them.[1]

It will be seen by these preliminary researches, as imperfect as they are, that two important probabilities were established. First, that certain microscopic beings—germs possibly identical in most of the cases, were found in the normal liquids and *tissues* of infectious Southern cattle that *always appear healthy*, and even in the *young before birth* (one was not older than four months after conception), as well as in the manures, surface soils and waters, etc., of grounds where the fever originates. Second, that this germ was absent in dry fodder, in well water, spring water, the subsoil (even the deep layer of sod being free). True, we

[1] The normal temperature of cattle is about 101° F.

were groping somewhat in the dark, and under disadvantages that made progress exceedingly slow, tiresome, and even somewhat doubtful to us, but it pointed to a possible clue. It furnished something like a tangible basis for further operations.

Questions 1, 2 and 3 of our objective task being partly answered, we could take up the remaining nine—*i. e.*, 4, 5, 6, 7, 8, 9, 10, 11, 12, with more hope.

A thirty acre pasture was secured on the Wabash Railroad between Centralia and Hallsville, in Boone County, Missouri. It was divided into two portions, and in one of them twenty-four pens were constructed with alley and chute leading directly to the railroad track, where, through the kindness of the Wabash management, experiment cattle were afterward loaded and unloaded at will. The pens were divided in two sets—one dozen numbered from 1 to 12 and one dozen lettered from A to L, inclusive. The strong board fences forming the enclosures were six feet high, and so closely framed that the cattle could not eat through although they could smell and even lick each other between the upper boards. The bottom boards came close enough to the ground to prevent eating underneath. The cattle were fed on the ground and watered in troughs purposely constructed. Their food was hauled to them from a distance.

*Experiments A.—Tests of the virulence of Texas cattle, and exposure of unprotected Northern Natives*—June 20th, received from the Texas Agricultural Experiment Station eighteen head of cattle supposed to be infectious. They had been shipped June 15th from Uvaldo, Uvaldo County, Texas, in a Street stable car. Before loading, the floor was covered three inches thick with sawdust and sand soaked with fifty gallons of water containing five pounds of corrosive sublimate (a quantity much larger than necessary), and a board was placed inside all around the car to prevent any droppings on the track from the floor.

After unloading the cattle at the experiment pens, the car was hauled to Hallsville and then to Columbia. Northern cattle *had access* to it and to the manure it contained; *not a case* of Texas fever occurred thereby. The cleanings of this car were also purposely tested on native Missouri cattle, and failed to produce the disease. Those eighteen head of cattle were distributed as follows: Pen 1, seven head; pen 2, eleven head. Three Missouri natives were put at once (June 20th) with the Texans in pen 1.

The eleven subjects of pen 2 were changed every second day from pen to pen until they reached pen 9 inclusive, then every day until pen 12 inclusive, which they left July 9th, making in all nineteen days in pens, and with the five days of travel making twenty-four days since they left Texas soil. June 21st, two native Northern cattle were put in pen 2. July 12th, we began to test the virulence of pens 3 to 8 inclusive where Texas cattle had been, by putting one head of Northern cattle in each. The results were as follows :

July 30th, cow in pen 8 died of Texas fever, exposed seventeen days. Virus of germs had been deposited thirty days before by the Texans. August 5th, bull in pen 5 seriously ill with the disease, exposed twenty-three days. The germs had been on the ground forty-two days at most. August 14th, cow in pen 1 died of Texas fever, exposed fifty-five days. The germs had been deposited by Texas cattle June 20th, or fifty-five days before. (This pen in shade.) August 15th, heifer in pen 7 died of same disease, exposed thirty-four days. Germs on the ground since June 28th, or forty-nine days. (Pen not shaded.) August 16th, heifer in pen 6 died, exposed thirty-five days. The germs were in the pen since June 26th, or fifty-one days. The exposed subjects in pens 2, 3 and 4 showed very feeble signs of illness. Nothing to be noticed by any one but the experienced observer. (Pen shaded part of day.) We had not enough cattle to test the virulence of pens 9, 10, 11 and 12, but the Texas stock, after leaving pen 8, *i. e.*, the 24th day after shipment from the South, seemed no longer infectious, as we mingled them with native stock in the pasture and no disease occurred thereby. I do not consider the test sufficient, however, on this particular point. June 21st, manure fresh from Texas cattle was deposited on the grass of pen G, and some was forwarded to the laboratory for analysis and tests. June 22d, 24th and 28th urine fresh and pure from Texas cattle was spread over the grass in pen F, and some of the same specimens were studied at the laboratory. Neither of these pens were at any time contaminated from any other source. August 6th, a native steer was placed in each pen. August 27th, or twenty-one days after exposure, the steer in pen F died of Texas fever, having contracted it from the urine deposited on the ground sixty-six, sixty-four and sixty days previously. The steer in pen G, where the manure was placed, showed so faint signs of the malady that only an experienced veterinarian could have observed anything unhealthy.

*Experiment A1.—First exposure of inoculated (or vaccinated) Northern stock*[1]—August 17th and 27th. After some of the deaths above recorded, and after pens 1 to 8 inclusive had been proven infectious, the disease and deaths recorded, they were thrown open together and in them were placed ten more head of Missouri cattle; six of which had been purposely inoculated with view to protect them; and four were not inoculated. The six inoculated all resisted the fever, whilst of the four not inoculated two died of Texas fever, one was seriously ill and recovered, and the other had no signs of serious sickness. These inoculations were made with weakened virus. More of this in the article on the subject of inoculation.

*Experiments A2.—Analysis of specimens from some of the above-mentioned Texas cattle and from natives that contracted the fever*—Fifteen fresh specimens of manure, urine, blood, bile, liver, spleens and kidneys of different apparently healthy Texas cattle were analyzed microscopically, and in all instances germs similar to those already described were found. The liver and bile in particular contained a great many. Twenty-six of the same kinds of specimens from all the Missouri cattle that died from Texas fever, or that were killed while suffering from it, also presented *always* the same germs, but in greater numbers. Great care was taken that the specimens in every case were procured right after death by fever, or right after killing the patient so affected. A special article on the germ of Texas fever will be found further. Inoculations of Missouri cattle with *strong* virus from infected organs of both *Texans* and *natives* produced Texas fever. That also will be better explained further on.

*Experiments B.—Tests of Arkansas cattle*—June 28th, 1889.—Dr. R. R. Dinwiddie (Veterinarian of the Experiment Station of Arkansas) shipped us from Helena, that State, in a car prepared as for the Texas shipment, twenty-five head of cattle supposed to be capable of conveying Texas fever. They arrived at our pens July 1st. The cattle had been marked and divided into several lots, and part of them had been dosed with antiseptic drugs, with a view to destroy the activity of the germs in the animals and

[1] Inoculation and vaccination are used here in the sense of protective inoculation with modified germs.

render them powerless to cause the disease on their arrival here. Unfortunately, the man who accompanied the stock failed to do his duty. On his way here he mingled the treated and untreated cattle together in the previously separated car, and then he left them by themselves during a large portion of the journey. The result was the stock could not be identified positively by the torn tags or for the want of marks; consequently the object desired in so treating the cattle antiseptically before shipment from Arkansas could not be intelligently sought. We therefore repeated in different pens the experiments made with the Texas cattle. In pen A five cattle were placed, in pen B five, in pen C four, and in pens J, K and L, opening in one another, eleven. Three of these Arkansas cattle, one in pen A and two in pens J, K and L, were sickly when they arrived and soon succumbed. In two of these there were marked lesions of Texas fever, and its specific germs were found in abundance. The other had a severe lung congestion, with mild lesions of Texas fever. These deaths are attributed to a combination of causes, chief of which were two days' neglect in feeding and watering by the negro who should have closely attended to the stock, as ordered by Dr. Dinwiddie. Right after the severe treatment that these animals had undergone, their bodies being impregnated with Texas fever germs, the best hygienic condition possible was necessary. July 30th, all of those Arkansas cattle were turned out of the pens. The same day five inoculated Missouri natives and two not inoculated were put in pens J, K and L. The inoculations had been made with virus of a nature which was doubtful at the time of operation. August 8th, one native cow was turned into pen C, not inoculated. August 11th, one native heifer was turned into pen B, not inoculated. August 20th, one native cow into pen E, not inoculated. The results were as follows: August 20th, in pens J, K, L one vaccinated animal, and next day two unvaccinated, presented symptoms of Texas fever. August 25th, heifer in pen A died of Texas fever, not inoculated. August 27th, one cow in pen C fatally ill from Texas fever, and killed purposely; not inoculated. Same date, one of the unvaccinated heifers in pens J, K, L died from Texas fever. Thus, of the five vaccinated or inoculated one was sick, but not one died; of the five unvaccinated exposed in different pens three died of Texas fever.

*Experiments B1.—Analysis of specimens of Arkansas and dis-*

*eased native Missourians*—Just as for Texas stock, specimens of various kinds were carefully investigated. Seventeen large specimens of manure, urine, blood, bile, liver and spleen from four different Arkansas cattle, and fifteen similar ones from natives that died from or were killed on account of Texas fever were collected fresh and subjected to searching microscopical examinations and bacteriological studies. In all of them the germs found in Texas cattle or diseased natives, as stated in Experiments A, etc., were noticed.

*Experiments C.—Tests with the second lot of Texas cattle*—These consisted of eighteen old cows shipped by Dr. Francis from the ranch of Col. Fulton, near Corpus Christi, August 6th or 7th. They arrived at our pens August 13th. The car had been previously prepared to prevent infectious droppings on the track, as for the other shipments. This lot was intended for the purpose of testing means to disinfect Southern cattle alive and render them harmless to Northern cattle. Some of them were to be untreated, to serve as witness cases. Unfortunately, Dr. Francis failed to secure the consent of the owner to this arrangement, and the cattle were shipped to us to be tested as to their individual virulence—the owner claiming that the cattle of his ranch never convey Texas fever. Our investigations not being carried on for any one ranch, however, this testing of one exclusive herd of cattle irrespective of a plan prearranged on an intelligent basis, and with respect to our financial condition and the welfare of the country at large, was not desirable. However, we did the best we could under the circumstances. The Fulton cattle were all placed in pens I and H, that had never been used for any purpose. September 9th, one yearling steer and one yearling heifer, natives, were put in the above pens. We had been unable to find, before this date, any stock at sufficiently low price for our extremely limited funds. October 19th, these two native cattle were turned out, no signs of fever having been detected by the superintendent. The thermometer was not used.

*Experiments C1.—Analysis of specimens from the Fulton cattle*—Samples of urine, manure, bile, blood, liver and spleen demonstrated the germs in a comparatively scarce quantity. Inoculations of a native heifer with small quantities of virus from these animals produced only a slight fever. By inoculation of a large quantity of liver pulp we did succeed in producing a good case of Texas fever in a native. These cattle seemed less virulent than others that we had. I am informed that the Fulton ranch is supplied with well and sulphur water. This suggests a valuable proposition, perhaps. Is it not possible that the peculiar water of the Fulton ranch is antagonistic to the life of the germ of Texas fever, and thereby modifies its virulence? We know the antise p tic properties of sulphur, for instance, and this is one of the very agents that we had instructed Dr. Francis to use to disinfect Southern cattle alive before shipping them North.

### DEDUCTIONS FROM THE EXPERIMENTS.

1st. That some of the germs found in our preliminary investigations of Southern soils, waters, manures, urine, bile, liver, etc., etc., and those found in the bodies of Southern cattle subsequently shipped to us and found in cases of Texas fever in Northern cattle, are identical.

2d. That the germs of Texas fever are to be found in all Southern cattle coming from infectious grounds, and not only in their manure, but in their blood, livers, spleen, kidneys, urine, etc., notwithstanding that they apparently remain healthy. The same is the case even with unborn calves, which are therefore naturally inoculated in the mother's womb.

3d. That the average period of exposure before the Texas fever appears North, in cattle exposed to the freshly deposited germs brought by Southern stock, is about thirty days; but this may vary much according to the sun heat and warm weather, which is a favorable condition, and cool or cold, which is unfavorable. The cattle in Pens 1, 2, 3, and 4, which were more shaded than the remainder, resisted longer and better.

4th. That the germs in fresh excretions of Southern stock remain apparently harmless between thirty and fifty days, and in a cool, shady place may remain thus much longer.

5th. That cattle exposed to either manure or urine from Southern stock may contract Texas fever, and that inoculation from pulp of liver or spleen of such subjects may produce it.

6th. That the germs must be taken into the body by the mouth or by inoculation, and that the disease is not conveyed by the breath of infected or infectious individuals.

7th. That protective inoculation may render the system of Northern cattle capable to better resist the action of the Texas fever germ.

8th. That sulphuretted water is perhaps inimical to the life of the germ and favorable to its modification and death.

SPECIAL EXPERIMENTS TO TEST PROTECTIVE INOCULATION.

*Note.*—Having discovered early in our experiments that Southern calves really have the germs of Texas fever in their system before birth, I became convinced that that was the first reason of the immunity of Southern stock against this affection. The young are actually inoculated—or vaccinated, to use a more familiar though not so accurate a term—in their mothers' wombs, and when dropped on the infectious Southern soil they are prepared to meet the virus that it contains just as well as a child vaccinated against smallpox can be safely exposed to that disease. The question that impressed me then was this: Why cannot the Northern cattle be artificially inoculated with the germs of Texas fever before exposing them South, or on any infectious ground, on the same basis that the Southern calves receive the germs from their mothers? I believed it possible, and we began experiments to that end. We soon found, however, that the proposition was not as simple as it appeared; obscure problems and discouraging failures met our efforts every hour of the day, and it required constant harassing microscopic researches and tiresome operations to keep at all in anything like a reasonable route. Inoculation after inoculation proved futile. We could scarcely detect anything like Texas fever symptoms in our first attempts to transmit it. Little by little, however, the darkness disappeared, the way became clearer, and at last we had in our possession, and partly under our control, the germ of the deadly plague, and we could cause this malady almost at will in a more or less virulent form. And at this writing we can cultivate the germs with comparative facility outside of the animal body, and then reproduce them in cattle by inoculation with such artificial cultures. Thus, after testing many kinds of virus and cultures from many sources, some of doubtful and others undoubted activity and purity; after inoculating by several methods once, twice, thrice or oftener in each case, with strong doses all at once, or several weak and strong at intervals; after trying gradual inoculation beginning with a mild virus and ending with a powerful one; after sacrificing many animals here and in the South, we feel glad; for we have, we think, partly conquered, and the people, our employers, may reap the great benefits that are sure to come if our future work gives the results promised.[1]

PROTECTIVE INOCULATION OF CATTLE SHIPPED TO TEXAS AND ARKANSAS.[2]

June 24th, six inoculated and six not inoculated head of cattle were shipped to College Station, Brazos Co., Texas, where they were put on pasture and attended to by Dr. Francis. The three kinds of virus used were not known to be positively reliable in any sense. Our experiments had not as yet given us a positive clue to its powers and effects. The elucidation of this double problem was one of the objects of this shipment. September 11th, shipped to the same place fourteen head of cattle, *i. e.*, ten inoculated and four not inoculated. They were also put on pasture. The atmospheric temperature was exceedingly warm.[3] The results obtained, as given by report of Dr. Francis, dated December 19th, 1889, were as follows:

*First Shipment.*—Of the six not vaccinated five died of Texas fever within three weeks, and the other was ill and recovered; of the six inoculated four died. Now, these inoculations had been practised once before leaving Columbia, and once in Texas, the virus being of a very *doubtful* nature, as before mentioned. Total per cent. of death of vaccinated stock 66⅔; the non-vaccinated stock 83 3 per cent.

*Second Shipment.*—Of ten vaccinated two died of Texas fever; of the four not vaccinated three died, and the others recovered after severe illness. Total death rate among the inoculated or vaccinated stock 20 per cent.; among stock not so protected 75 per cent. The origin of the virus used for inoculation was manure, liver, blood, etc., and had been cultivated or prepared artificially. It will be seen that the last shipment was more successful than the first, although far from satisfactory. It remains to be said that the stock thus treated and exposed that did resist Texas fever, did not do as well as stock of Southern origin kept on the same grounds. The subjects remained poor for awhile. July 5th, four inoculated cattle and four not inoculated were sent from Columbia to Helena, Arkansas, and there exposed on the grounds, under the care of Dr. Dinwiddie. August 9th, eleven

[1] I give in this bulletin our failures as well as successes.

[2] Inoculation and vaccination here mean the same.

[3] The inoculated cattle of these shipments were Missouri natives.

head, six inoculated and four not inoculated, were shipped to the same place. Four different kinds of virus of unknown strength and power had been used as a logical deduction of laboratory studies, with a view to test the chances of finding a protective kind. The result of the two experiments was that *one died of Texas fever on the cars, the effect of inoculation ;* twelve succumbed on the grounds (possibly two of these died of anthrax), and six are now living. From the latest report of Dr. Dinwiddie, I infer that the latter *were all* inoculated stock. However that may be, the results in a general way could not be considered encouraging. But when it is explained that nearly all the inoculated stock that survived these four shipments to Texas and Arkansas had been treated with virus of *similar origin and nature*, it gives hope that some good will come of this particular kind. Dr. Dinwiddie's report of much faithful work shows 100 per cent. of death of non-vaccinated ; 75 per cent. vaccinated.

### INOCULATION IN HOUSTON, TEXAS CO., MO., AT THE KANSAS CITY STOCK YARDS, AND AT OUR EXPERIMENT PENS.

July 19th, an outbreak of Texas fever of extensive character was reported at Houston, Texas Co., Mo. Dr. B. R. Harmon, then of the State Veterinarian's Laboratory, was deputized to attend to the matter. He remained ten days on the grounds and found that several car loads of Southern cattle had been brought into the county April and July, and scattered over the unfenced lands to roam at large. Some fourteen head of the natives among them had died, and several were sick with Texas fever. Dr. Harmon practiced inoculation with an artificial preparation of the germs of Texas fever on thirty-one head of cattle. They were left exposed with the Southern cattle on the infected grounds. All remained healthy, but it was reported that several of the unprotected cattle left with them afterward contracted the malady and some died. This was only an incidental experiment during our regular investigations, and is not of itself sufficient evidence.

*At the Kansas City Stock Yards.*—The work here was conducted by Dr. H. B. Adair, Deputy State Veterinarian, a skillful surgeon, and witnessed by P. D. Etue, Esq., editor of the *Kansas City Live Stock Indicator*, who manifested a great deal of interest in the undertaking and sacrificed much time to it. Dr. J. A. Walrath, of the Bureau of Animal Industry, was present also on several occasions. August 10th, six native heifers were placed in lot 13, block 20, which was free from contagion. They had never been exposed to Texas fever and were in good health. Temperature of the six averaged 101 1-5° F., before inoculation. Heifers Nos. 1, 2 and 3 were inoculated with an artificial culture of Texas fever germs furnished by me. August 14th, heifers Nos. 4, 5 and 6 were inoculated as directed by me with spleen and liver pulp taken from a case of Texas fever soon after death. This virus, unfortunately, contained some septic germs and caused some little trouble. During the next six days after inoculation the temperature of the first lot inoculated rose to an average of 102 4-5° F., and that of the second lot rose to an average of 104° F. August 17th, Nos. 1, 2 and 3 were re-inoculated with the same kind of cultivated germs, but of greater virulence. During the five days following this inoculation the temperature rose to an average of 103 2-5° F. Thus the first three were inoculated twice and the second three only once. August 22d, both lots were examined and the temperature for each was as follows: *First lot* (inoculated twice), No. 1, 102 2-5° F.; No. 2, 103 2-5°; No. 3, 103 1-5°; *Second lot* (inoculated once), No. 4, 105 1-5°; No. 5, 103°; No. 6, 105 2-5°.; Nos. 4 and 5 appeared sick. August 24th, the temperature had abated in all six to an average of 103° F. The inoculation with spleen and liver pulp had produced swellings and slight abscesses at the points of inoculation —the brisket. This date all the *vaccinated animals*, together with three others, *Nos. 7, 8 and 9, not vaccinated*, were driven across the stock yards bridge to the Kansas side, and there placed successively in lots 12, 6 and 5, block 32, occupied some time previously by Southern cattle that had proven their power of conveying Texas fever.

The final results of this experiment are given by Dr. Adair as follows: "Case No. 3, *inoculated*, died of Texas fever; case No. 4 died of septicæmia induced by the *inoculation*. Neither the symptoms nor the lesions found in post-mortem examination indicated Texas fever. (Microscopic investigation verified this diagnosis.) The remaining four, all vaccinated, remained healthy. No. 7, not vaccinated, died early of Texas fever. Nos. 8 and 9, also unprotected, both contracted it severely, but finally recovered. Thus of the *six vaccinated*, *only one* contracted Texas fever, whilst the *three unvaccinated subjects all* became affected,

THE FOLLOWING TABULATION SHOWS THE MOST IMPORTANT POINTS OF THESE EXPERIMENTS AT A GLANCE.

| Case. | Inoculated or not, and date of Inoculation. | Origin and activity of virus used for inoculation. | Average temperature of animals from 1st to 5th day. | Lowest and highest temperature of the cattle, from the 10th to the 20th day. | Results after twenty days of exposure and later. |
|---|---|---|---|---|---|
| A. | No. | | 101 3-5° F. | 102 to 103° F. | Died of Texas fever. |
| B. | No. | | 101 | 102 3-5—103 3-5 | Very sick, but recovered. |
| C. | No. | | 101 | 101—102 | Apparently well, always. |
| D. | No. | | 102 | 102—104 | Died of Texas fever. |
| E. | Yes ; July 20th. | Spleen—weak. | 103 | 103—104½ | Fever diminished. |
| F. | Yes ; July 20th. | Spleen—stronger. | 104 | 103 2-5—103 | Not sick to show symptoms. |
| G. | Yes ; July 22d. | Spleen—weak. | 102 | 104—106 | Fever declined. |
| H. | Yes ; July 26th. | Artificial cult.—strong. | 104½ | 103—102 3-5 | Not sick, apparently. |
| I. | Yes ; July 26th. | Artificial cult.—stronger | 105 | 104—103 | Fever from inoculation, not sick from exposure. |
| J. | Yes ; July 26th. | Liver—very strong. | 105 | 105—104 3-5 | Sick from inoculation, not from exposure. |

Thus of the four not inoculated before exposure, two died of Texas fever, one was very seriously ill, and the other remained apparently well. Of the six inoculated none died, two were not sick, and the remainder sick mostly from inoculation, as explained in the result column. The subject of protective inoculation will be especially treated further.

and one died. During the fifty-five days of this test the weather was very warm. The cattle were kept in open pens exposed to the sun's rays. Their solid food consisted of hay only, and sometimes it was not good ; consequently all of them became thin, and the trial was a most severe one."

*At our Experiment Pens.*—As mentioned already, inoculated and unvaccinated cattle were put in infectious pens 1 to 8 inclusive, August 17th and 27th.

INOCULATION OF CATTLE SHIPPED TO PRIVATE INDIVIDUALS IN THE INDIAN TERRITORY AND TEXAS.

In November, 1890, after we had learned the lessons of the preceding experiments, an agreement was entered into between Dr. A. W. McAlester, of Columbia, Mo., and the writer, to the effect that forty head of high grade or thoroughbred Hereford, shorhorn and other cattle were purchased by the doctor for his cousin, J. J. McAlester, of McAlester Station, Indian Territory, and that we inoculated them, and shipped them to the latter place, where they were scattered over infectious grounds on which the death rate by Texas fever had always been so severe, even during the greatest part or the whole of mild Winters, that Mr. McAlester, after repeated and fatal trials, had given up the importation of Northern stock. The process of inoculation was gradual, giving twenty drops of virus to each head every third day until four inoculations had been practised. Each successive virus was stronger than the preceding, and the last was very virulent. The animals were shipped next day after the last inoculation, and were unloaded at McAlester Station, December 1st, 1889, about two days after shipping. During the inoculation process the temperature rose from the average normal of 101 2-5° F., to a fever averaging 103.° When they reached the Territory this fever had declined some, as proven by Dr. Grubbs, who registered the temperature for Mr. McAlester. The results of this trial are given here in the language of the purchaser himself.

"Of the forty head bought and inoculated by you *only* one died of Texas fever some time since. A neighbor received from Missouri two cows and two yearlings not vaccinated and placed them on practically the same ground at about the same time that mine were put on pasture and every one of his died of Texas fever. I am of the opinion that this vaccination with the treatment that you prescribed will save nine-tenths of the cattle, particularly the young stock shipped to this climate. This has been an unusually warm Winter. Most of the cattle are now scattered over the country and all doing very well but one, and he is doing fairly."

The usual death rate of Northern cattle in the Indian pastures where these were exposed has always averaged over 50 per cent., and continued its ravages in the Winter, especially mild Winters.

### DEDUCTIONS FROM EXPERIMENTS.

1. That if Texas fever is, as has been written, of the obscure nature of malaria in man, it is nevertheless surely inoculable, and is due to an appreciable germ.

2. That this germ or microbe, even when cultivated artificially, may be inoculated to cattle with dangerous effects.

3. That susceptible cattle, (*i. e.* of the North) may be purposely inoculated with virus of Texas fever, and thereby placed in the same relation with that disease as are the cattle born South which become primarily inoculated from their mothers.

4. That one single inoculation, mild or powerful, is not a sufficient protection against Texas fever, and that the virus must be of certain power and prepared purposely with the greatest care and by scientific methods.

5. That inoculation with too strong virus, causing high fever, or positive serious signs of Texas fever, is not a sure protection, but instead is a dangerous practice and may kill stock directly, or cause such damage in the system that when exposed to the virus when thus suffering or soon thereafter, the subjects gradually get worse and die—*never* having regained their strength.

6. That periodical inoculation, beginning with a mild virus and ending with a stronger, is more rational as being more like the natural process of inoculation of Southern calves, and is in a large degree a protection againt Texas fever.

7. That possibly the safest method of inoculation is to complete it on Southern soil after shipment, providing that there be no expo-ure to the germs during the period of inoculation, particularly at the beginning.

8. That the best results of inoculation thus far are obtainable when the greatest care and attention is given to the inoculated stock before and after it is exposed.

[TO BE CONTINUED.]

